# Autologous Free Dermal-Fat-Fascial Graft for Parotidectomy Defects: A Case Series

**DOI:** 10.1177/0003489421999542

**Published:** 2021-03-04

**Authors:** Aliasghar A. Mianroodi, Sadaf Mohtashami, Nahir Romero, Andrew Fuson, Arjun Joshi, Nader Sadeghi

**Affiliations:** 1 Division of Otolaryngology-Head and Neck Surgery, George Washington University, Washington, DC, USA; 2 Department of Otolaryngology-Head and Neck Surgery, McGill University, Montreal, QC, Canada

**Keywords:** parotidectomy, facial contour, Frey’s syndrome, free dermal-fat-fascial graft, gustatory neuralgia

## Abstract

**Background:**

Frey’s syndrome and facial asymmetry from loss of parotid tissue are long-term sequelae of parotid surgeries causing significant morbidity. Various techniques have been used to fill the parotidectomy defect, preserve facial contour symmetry, and prevent Frey’s syndrome. Free dermal-fat-fascial graft (DFFG) is one such technique; however, its use is largely undocumented in the literature. In this case series, we investigate the efficacy of free DFFG in reconstructing parotidectomy defects at 2 tertiary care centers.

**Materials and methods:**

Medical records of 54 patients who underwent primary parotidectomy and immediate reconstruction with autologous abdominal free DFFG by 2 surgeons in George Washington University Hospital and McGill University Health Centre between 2007 and 2019 were collected prospectively. Patients responded to 2 questionnaires addressing postoperative outcomes.

**Results:**

Fifty-four patients were included; 32 superficial parotidectomies and 22 total parotidectomies were performed for 39 benign and 15 malignant tumors. Thirty-seven patients could be reached. Out of 37 patients who responded to the first questionnaire, 59% (22) reported complete facial symmetry, 27% (10) reported mild hollowness, and 14% (5) reported mild fullness. None declared noticeable hollowness or fullness. While 81% (30) did not experience Frey’s syndrome, 5.4% (2) experienced mild symptoms without disability, and 13.5% (5) experienced debilitating symptoms. Out of 37 patients, 8 patients responded to a second questionnaire addressing the outcome of the abdominal graft donor site. In regard to the donor site, 87.5% (7) were satisfied or very satisfied from its cosmetic appearance, 75% (6) were not bothered by its cosmetic appearance, and 87.5% (7) had no discomfort at the graft donor site. Patients did not report any other symptom at the graft donor site.

**Conclusion:**

In this large series of total parotidectomies including malignant pathologies, autologous abdominal free DFFG effectively prevented Frey’s syndrome and preserved facial cosmesis in most patients.

## Introduction

Frey’s syndrome and facial asymmetry from loss of parotid tissue are common long-term sequelae of parotidectomy. Interposition of a graft between the skin flap and the parotid bed can prevent these sequelae as it acts as a barrier preventing aberrant innervation of the skin sweat glands by auriculotemporal nerve fibers while adding bulk to the parotid bed, filling the deficit resulting from parotidectomy.^1-5^

The exact incidence of Frey’s syndrome is difficult to determine as many patients remain unaware of their symptoms or may not report them. Also, administering and quantifying the objective Minor’s test is difficult.^6^ In the absence of interposition grafting, the incidence of Frey’s syndrome is 43% to 100% if tested objectively and 12% to 62% if measured subjectively based on symptoms reported by patients such as unilateral hyperhidrosis, redness, and flushing with stimulus.^7^ Both subjective and objective incidence of Frey’s syndrome have been shown to be significantly decreased if interposition grafting is used.^8-10^

There are many techniques for interposition grafting such as sternocleidomastoid (SCM) flap, sub-superficial musculoaponeurotic system (SMAS) flap, platysma flap, temporoparietal fascial flap, autologous tissue flaps with microvascular repair, allogenic dermis, implantable synthetic materials, free fat grafts, and dermal fat grafts.^5,9–17^

Each technique has its unique advantages and disadvantages. The SCM flap is well vascularized, and does not require further skin incision; however, it carries a risk of spinal accessory nerve and facial nerve (FN) injury, and SCM atrophy. The temporoparietal fascial flap requires a longer incision and carries risk of FN injury, temporal atrophy, zygomatic fullness, and alopecia. In addition, it does not provide adequate bulk for deep defects. Allogenic human dermis does not require an additional surgical site; but, it is expensive and has been associated with an increased duration of suction drainage, and an increased incidence of sialocele, seroma, and salivary fistula formation.^18,19^ Similarly, implantable synthetic materials such as vicryl and expanded polytetrafluoroethylene mesh have been associated with increased incidence of sialocele and implant extrusion.^6^ While all of these interposition grafts have been effective in decreasing the incidence of Frey’s syndrome, they have a minimal effect on post-parotidectomy cosmesis with inadequate bulk to restore the facial contour.

In contrast to the above-mentioned strategies, fat grafts, reported as early as 1893, were found to be effective in preserving facial cosmesis.^20^ Using fat as a reconstruction material has many advantages: it resists infection, remains supple, does not elicit a foreign body reaction and accommodates facial development and growth in the pediatric population.^21^ One of the most robustly reported methods of fat autograft reconstruction is abdominal free fat grafting which has been shown to be effective in filling head and neck defects including parotidectomy defects across different surgical techniques.^5,8,10,20-28^ However, free fat grafts suffer from an unpredictable and significant resorption rate. The inclusion of dermis with a free fat graft may aid in revascularization of the transplanted fat, decreasing resorption.^22,26^ The dermal fat graft is a well-established technique, recognized for its utility in Facial Plastic and Reconstructive Surgery and complex head and neck reconstructions.^28,29^

In this case series we are reporting on parotidectomy defects reconstructed primarily with autologous abdominal free DFFG in 2 tertiary care centers. Compared to the existing literature, our findings represent the largest series of free DFFG in the context of post-parotidectomy reconstruction, as well as the largest series to include total parotidectomies, parapharyngeal resections, and malignant pathologies.

## Materials and Methods

### Patient Selection

A prospective collection of the medical records of patients who underwent primary parotidectomy and immediate reconstruction of post-ablative defects with DFFG between 2007 and 2019 was conducted. All surgeries were performed by 2 surgeons using the same technique. Patients with recurrent parotid tumor, prior parotid radiation, and patients who underwent reconstruction using free flap for massive defects with extensive loss of overlying skin were excluded. Both superficial and total parotidectomies were performed with or without FN preservation. Out of 229 patients who underwent parotidectomy, 54 met the inclusion criteria.

Chart review was conducted to collect any missing data from the prospective database. Data included patient demographics, type of ablative procedure, neoplasm size and volume, final pathologic classification of the resected neoplasm, adjuvant radiotherapy, facial contour, hyperhidrosis, flushing, gustatory neuralgia, and subjective morbidity at the abdominal graft donor site. Data analysis was done using chi-square test in Minitab 15.

### Patients’ Reported Outcome

In order to assess patients’ satisfaction from cosmetic and functional outcomes of the reconstruction, the patients were asked to respond to 2 questionnaires by telephone or in person. These questionnaires are not validated; however, they had been used in the literature for patient-reported outcomes. To avoid interpretation bias by treating surgeons, cosmesis was assessed by the patients themselves using a 5-point Likert scale (1-very dissatisfied, 5-very satisfied). The results of the patients’ survey were collected, tabulated, and reported in the study (Tables 1 and 2).

**Table 1.** Patient Reported Outcome Measures and Distribution.

Patient reported symptom	Outcome	No (%)
Facial contour	Symmetric	22 (59)
	Operated side mildly hollow	10 (27)
	Operated side noticeably hollow	0
	Operated side mildly too full	5 (14)
	Operated side noticeably too full	0
	Total	37 (100)
Gustatory neuralgia	Not at all	34 (92)
	Yes, not causing any problems	2 (5.4)
	Yes, sometimes a problem	1 (2.7)
	Yes, always a problem	0
	Total	37 (100)
Facial flushing	Not at all	35 (94.6)
	Yes, not causing any problems	1 (2.7)
	Yes, sometimes a problem	1 (2.7)
	Yes, always a problem	0
	Total	37 (100)
Frey’s syndrome (facial hyperhidrosis)	Not at all	30 (81)
	Yes, not causing any problems	2 (5.4)
	Yes, sometimes a problem	1 (2.7)
	Yes, always a problem	4 (10.9)
	Total	37 (100)

**Table 2.** Patient Reported Outcome Measures and Distribution Regarding the Graft Donor Site.

Patient reported symptom	Outcome	No (%)
Satisfaction of the cosmetic appearance of the graft donor site	Very satisfied	3 (37.5)
	Satisfied	4 (50)
	Dissatisfied	1 (12.5)
	Very dissatisfied	0
	Total	8 (100)
Bothered by the cosmetic results of the graft donor site	Not at all	6 (75)
	Yes, mildly	2 (25)
	Yes, moderately	0
	Yes, extremely	0
	Total	8 (100)
Discomfort in the graft donor site	Not at all	7 (87.5)
	Yes, mildly	1 (12.5)
	Yes, moderately	0
	Yes, extremely	0
	Total	8 (100)
Any symptoms from the graft donor site	Yes, what symptoms do you have?	0
	No	8 (100)
	Total	8 (100)

### Surgical Technique

**Figure 1.**
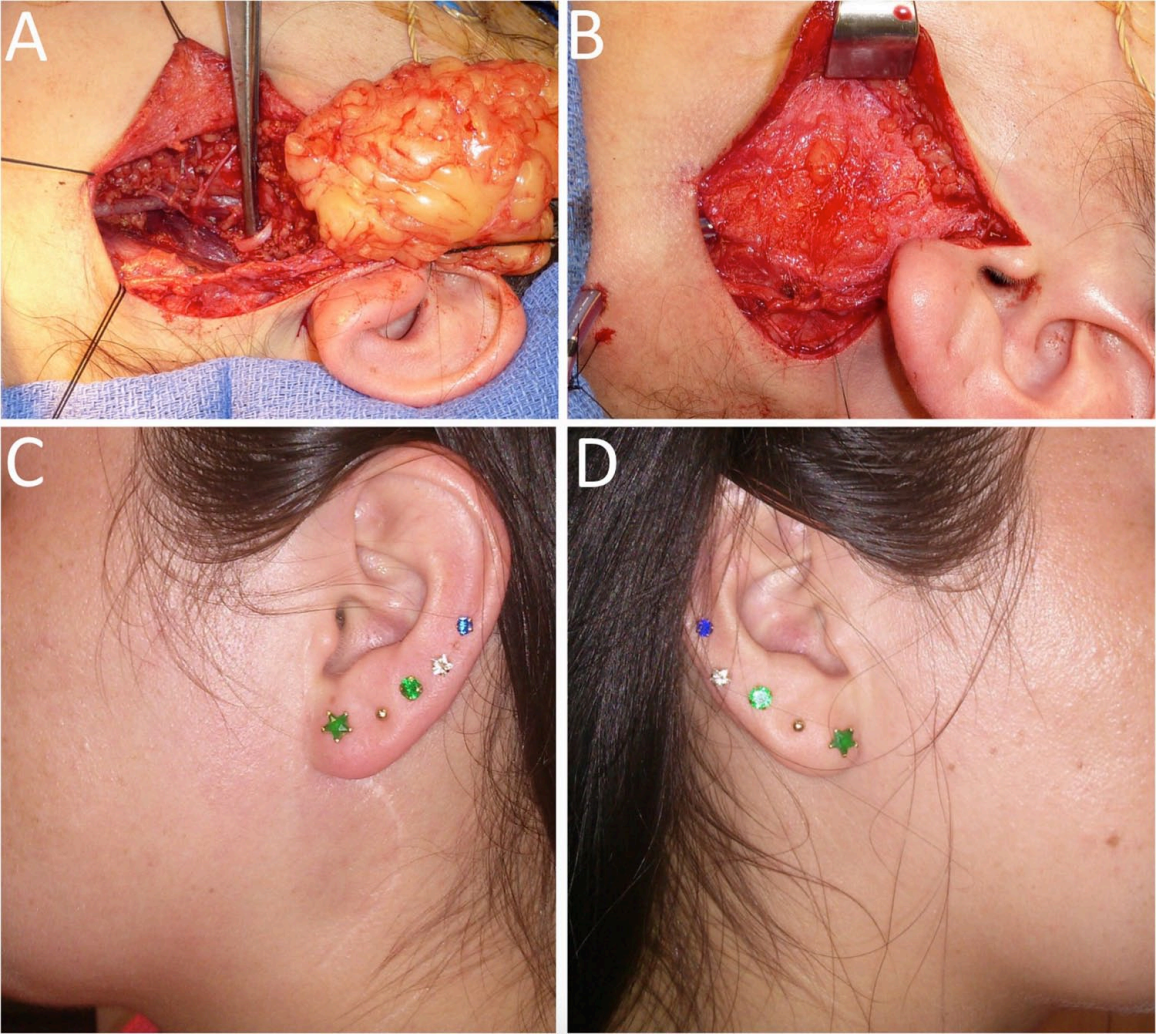
Dermal-fat-fascia graft at 5 years following reconstruction for a left lateral parotidectomy defect for a pleomorphic adenoma. (A) Parotidectomy defect with DFFG next to it. (B) DFFG with dermis sutured in place. (C) Facial contour of the operated side. (D) Facial contour of the non-operated side.

Immediately following parotidectomy and measurement of the size of the resected specimen, the DFFG is harvested from the abdomen; the suprapubic area or left lower quadrant is generally used. An elliptical area is mapped out on the donor site corresponding to the size of the defect. The area is de-epithelialized in situ while preserving the dermis. Incisions are then made through the dermis and the underlying adipose tissue for adequate thickness. The graft containing dermis and underlying fat (DFFG) is then harvested and trimmed down to adequate size matching the defect. The graft is taken 20% to 30% larger than the parotidectomy specimen as the graft will always undergo some degree of resorption postoperatively. The graft is then placed in the parotid bed with the dermis on the superficial side, and the dermis is sutured all around onto the SMAS using 3-0 vicryl suture (Figure 1). This allows precise positioning of the graft, preventing any migration while restoring the continuity of the SMAS. The skin flap that was raised for parotidectomy is then sutured back in standard fashion, covering the DFFG.

## Results

A total of 54 patients met the study inclusion criteria. The mean age of the inclusion group was 43.4 years (range 20-70, SD 11.2). Thirty patients were females (55.5%) and 24 patients were males (44.5%). The average follow-up was 11.8 months (range 0.2-59.2 months, SD 9.7) (Table 3).

**Table 3.** Patient Demographics, Types of Surgery, Tumor and Specimen Volume.

Patients demographic data	Number	Percent
Male	24	44.50
Female	30	55.50
Mean age	43.4 y (range 20-70, SD 11.2)	
Mean follow up	11.8 mo (range 0.2-59.2, SD 9.7)	
Surgery and tumor characteristics	Number	Percent
Lateral parotidectomy with FN spared	32	59.30
Total parotidectomy with FN spared	17	31.50
Total parotidectomy with FN sacrificed	3	5.50
Total parotidectomy with parapharyngeal resection	2	3.70
Mean specimen volume and SD (ml)	45.65 ± 44.6	
Mean tumor volume and SD (ml)	13.36 ± 12.8	

Thirty-two patients had superficial parotidectomy and 17 had total parotidectomy with the FN preservation (Table 3). The 3 patients with FN sacrifice had malignant neoplasm. Two patients had total parotidectomy with parapharyngeal resection for parotid neoplasms extending into the parapharyngeal space. Five patients with malignant neoplasm received postoperative radiotherapy. Out of 54 patients, 39 had benign tumors with pleomorphic adenoma being the most common type and 15 had malignant tumors with mucoepidermoid carcinoma being the most common type (Table 4).

**Table 4.** Distribution of Final Parotid Gland Pathology Following Parotidectomy and Dermal Fat Fascial Graft Reconstruction.

Tumor		Number	Percent
Benign	Pleomorphic adenoma	35	65.0
	Basal cell adenoma	1	1.85
	Schwannoma	1	1.85
	Benign cystic lesion	1	1.85
	Lymphoepithelial lesion	1	1.85
Malignant	Mucoepidermoid Ca	6	11.0
	Adenocarcinoma	2	3.7
	Mammary analog secretory carcinoma of salivary gland	2	3.7
	SCC	1	1.85
	Salivary duct carcinoma	1	1.85
	Papillary cystadenocarcinoma	1	1.85
	Oncocytic carcinoma	1	1.85
	Adenoid cystic carcinoma	1	1.85
Total		54	100

Out of 54 patients, 37 were seen in the surveillance visit or reached by telephone for survey completion. They responded to the first questionnaire (Table 1). In regard to satisfaction with the facial contour, 59% (22) reported complete facial symmetry, 27% (10) reported mild hollowness, and 14% (5) reported mild fullness. None declared noticeable hollowness or fullness. In regard to Frey’s syndrome, 81% (30) did not experience any clinical symptoms, 5.4% (2) experienced mild symptoms without disability, and 13.5% (5) experienced debilitating symptoms.

**Figure 2.**
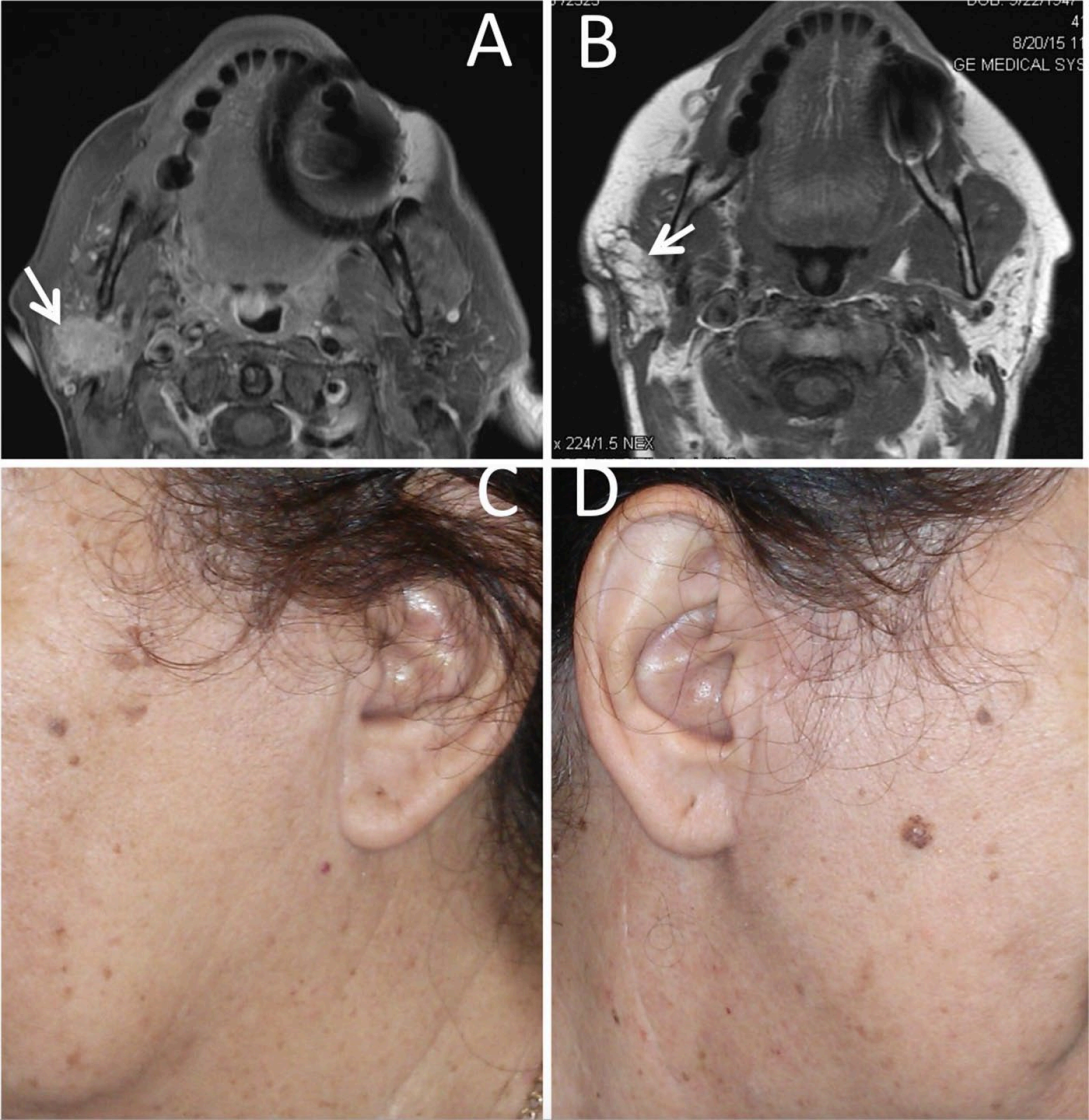
Dermal-fat-fascia graft at 1.5 years following reconstruction for a right lateral parotidectomy defect for a mammary analog secretory carcinoma. (A) MRI imaging showing the carcinoma prior to parotidectomy. (B) MRI imaging showing the graft after parotidectomy. (C) Facial contour of the non-operated side. (D) Facial contour of the operated side.

**Figure 3.**
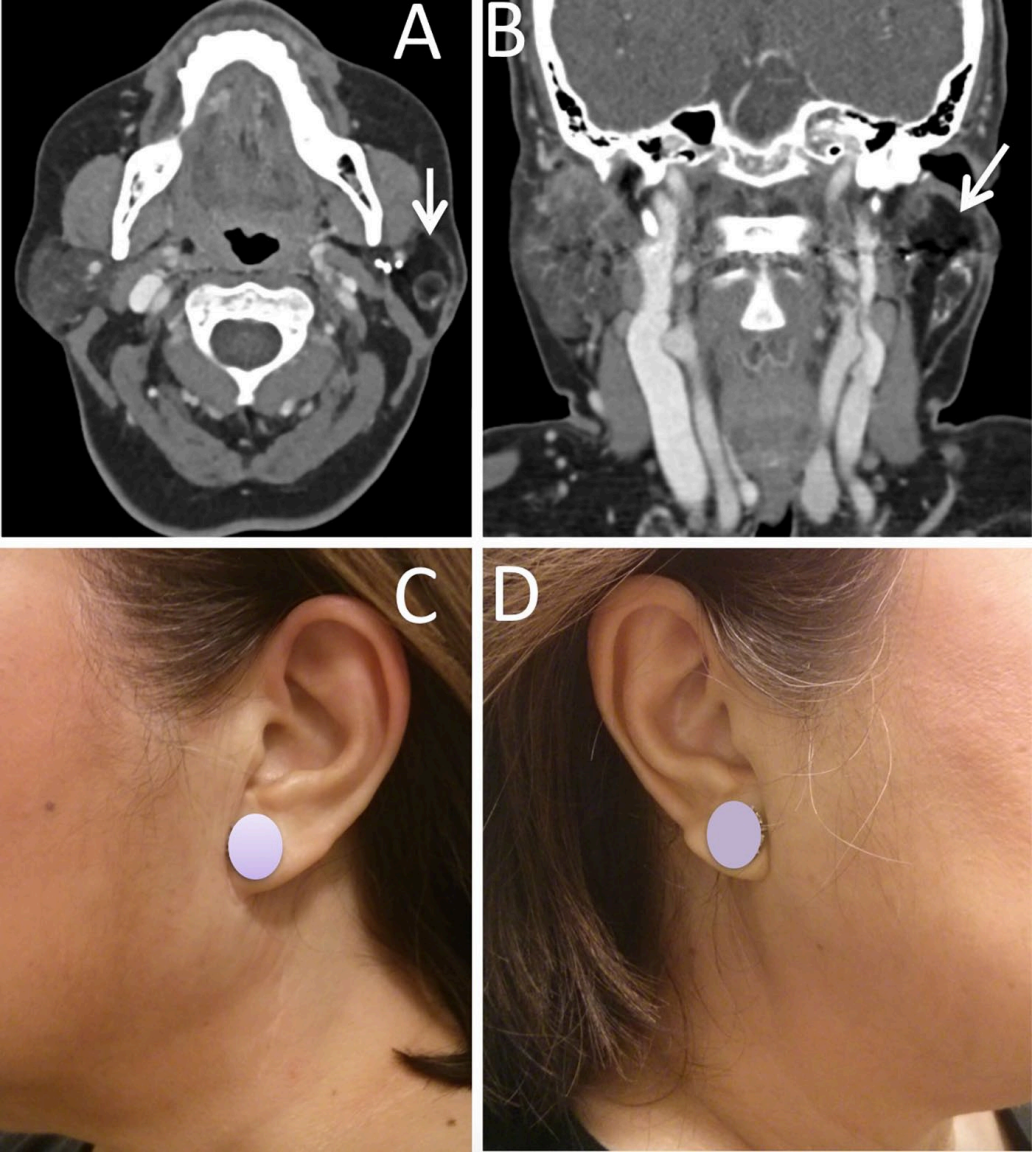
Dermal-fat-fascia graft at 4.5 years following reconstruction for a left lateral parotidectomy defect for a pleomorphic adenoma in axial (A) and coronal (B) CT scan imaging. (C) Facial contour of the operated side. (D) Facial contour of the non-operated side.

Figure 1 belongs to a female patient with left lateral parotidectomy for a pleomorphic adenoma who reported mild fullness of the operated side. On initial examination by the treating surgeon, this patient had full facial symmetry; however, there was some stiff nodularity due to graft atrophy which was managed by Kenalog injection into the graft twice. After injection, the graft became soft and 1-year post surgery, this patient had normal and symmetric facial contour and did not report any symptom of Frey’s syndrome. Figure 2 shows another example of a patient who reported mild fullness of the operated side compared to the non-operated side. Figure 3 shows an example of a patient who reported complete symmetry of the facial contour.

Besides the 2 main outcomes of the study, Frey’s syndrome and facial contour symmetry, the first questionnaires also addressed gustatory neuralgia and facial flushing. In regard to gustatory neuralgia, 92% (34) did not experience it, 5.4% (2) experienced mild symptoms without disability, and 2.7% (1) experienced debilitating symptoms (Table 1). In regard to facial flushing, 94.6% (35) did not experience it, 2.7% (1) experienced mild symptoms without disability, and 2.7% experienced debilitating symptoms (Table 1).

Out of 37 patients who responded to the first questionnaire, 8 patients responded to the second questionnaire addressing the outcome of the abdominal graft donor site (Table 2). In regard to the donor site, 87.5% (7) were satisfied or very satisfied from its cosmetic appearance, 75% (6) were not bothered by its cosmetic appearance, and 87% (7) had no discomfort at the graft donor site. Patients did not report any other symptom at the graft donor site.

Postoperative radiotherapy was shown to not have an impact on Frey’s syndrome (*P*-value = .38) and facial contour (*P*-value = .271).

## Discussion

This series demonstrated excellent cosmetic and functional results in patients who underwent parotidectomy and immediate reconstruction with autologous abdominal free DFFG. The DFFG is durable, easily harvested with the size and thickness adjusted to match the defect and features minimal donor site morbidity. It fills the preauricular hollowness and acts as a mechanical barrier to the aberrant reinnervation that causes Frey’s syndrome.

The rate of malignancy in this study, 28% (15/54), is similar to rates of malignancy reported by Baum et al^27^ (4/19 = 21%). Furthermore, similar distributions of final pathology results have been reported in the literature with pleomorphic adenoma being the most common tumor of the parotid gland, as was the case for 65% of patients in this study Similarly, the rates of Frey’s syndrome and facial symmetry found in this study are comparable to the existing rates in the literature using DFFG. In this study, rates of cosmetic dissatisfaction and subjective morbidity were low, with 59% of the patients reporting symmetric facial contour, 81% having no symptoms of Frey’s syndrome, 94.6% with no facial flushing, and 92% not experiencing gustatory neuralgia.

Honeybrook et al^28^ assessed the efficacy of free DFFG in reconstruction of head and neck defects in 62 patients including 22 parotid reconstructions. According to their study, 81.2% (26/32) of the patients were satisfied from the cosmetic results and 98.3% (61/62) did not experience Frey’s syndrome. Baum et al^27^ reported no subjective incidence of Frey’s syndrome in 19 patients undergoing parotidectomy including 17 superficial and 2 total parotidectomy reconstructed with DFFG. They also reported a 10% (2/19) rate of permanent overcorrection and 10% (2/19) rate of undercorrection of the parotid defect leading to fullness and hollowness of the surgical defect, respectively. Harada et al^8^ reported a 14% (1/7) incidence of subjective Frey’s syndrome and a 14% (1/7) incidence of overcorrection of the surgical defect in patients undergoing superficial parotidectomy for benign disease reconstructed with DFFG. Chandarana et al^10^ found a 12% incidence of objective Frey’s syndrome following superficial parotidectomy for benign disease reconstructed with DFFG. Davis et al^21^ reported that 33% (7/21) of their patients required a repeat procedure or had poor cosmesis following DFFG reconstruction.

This series presents a larger proportion of total parotidectomies (20/54), parapharyngeal resections (2/54), and malignant pathology (15/54) than previous reports using DFFG for parotid defects. This may have influenced both the incidence of Frey’s syndrome and cosmetic outcomes. As the size of the defect and the extent of parotidectomy increases, the subjective incidence of Frey’s syndrome and facial asymmetry may increase. Two studies found that in absence of reconstruction, the rate of Frey’s syndrome is 47% in total parotidectomies, 17% in superficial parotidectomies, and 3% in extracapsular dissections.^30,31^

This cohort reported good patient-reported outcome with respect to the abdominal graft donor site; the majority of patients were satisfied with the cosmetic appearance (87.5%), were unbothered by the cosmetic appearance (75%) and had no discomfort at the graft donor site (87%). Similarly, Honeybrook et al did not report any complication at the abdominal graft donor site and all patients were satisfied from the donor site cosmetic results. Chan et al^5^ also did not report any complication at the abdominal graft donor site and Baum et al^27^ reported hypertrophic scar, hypoesthesia, itching, recurrent pain, and hypersensitivity in 16% (3/19) of the patients. None of the patients in our cohort had any of these symptoms. One patient who reported discomfort at the graft donor site commented on stretching of the skin when performing certain physical maneuvers. The patients who reported only being satisfied, as opposed to very satisfied, and the patients who reported being bothered by the cosmetic results commented on the large size of the scar and the discoloration of the scar.

Graft resorption is the main concern when free DFFG is used for reconstruction. Resorption can lead to facial asymmetry and poor cosmesis. It has been suggested that dermal fat grafts exhibit improved survival compared to free fat grafts, as the intact dermal vascular plexus may encourage adipocyte survival. In an animal study, free fat, dermis fat, and dermal-fascia-fat were compared; it was found that dermal-fascia-fat graft undergoes more angiogenesis and collagen synthesis in comparison to other groups.^32^ Chandarana et al^10^ studied DFFG survival at 1- and 6-months following reconstruction of a parotidectomy defect with or without a platelet-rich plasma solution. The resorption rate was 66% to 82% in the control group, and 30% to 66% in the platelet-rich plasma group.^6^ This rate is slightly higher than reported rates by other authors because most studies recommend using a graft that is 20% to 40% larger than the parotidectomy defect.^8,11,13^ In this series, the grafts were consistently overcorrected by 20% to 30%, which we believe contributed to the fact that no one in our series reported noticeable hollowness or fullness.

Besides resorption, other concerns when using free DFFG are fat liquefaction leading to seroma, wound infection, and wound dehiscence. Varying rates of complications have been reported for free DFFG reconstruction. Baum et al^27^ reported a complication rate of 21% (4/19); 2 hematomas and 2 seromas. Harada et al reported no complications, and Chandarana et al reported 12.5% (2/16) complication rate due to seromas.^8,10^ One patient in this cohort developed wound infection necessitating operative debridement of the DFFG. Another patient developed a nodularity of the graft, which resolved completely after Kenalog injection as discussed previously. This patient reported excellent facial contour and no symptom of Frey’s syndrome 1-year post-parotidectomy.

In this study, postoperative radiotherapy was shown to have no impact on both cosmetic and functional outcomes post parotidectomy reconstructed with DFFG. However, only a small number of patients (5 patients) received radiotherapy. In order to claim that postoperative radiotherapy has no detrimental impact on outcomes, a larger number of patients with postoperative radiotherapy is needed.

Similarly, Honeybrook et al^28^ did not find any differences in cosmetic outcomes when comparing radiated and non-radiated cohorts who underwent DFFG reconstruction for head and neck defects including parotidectomy.

Historically, there have been some hesitations in reconstructing parotidectomy defects for malignant pathology as malignant gland may require postoperative radiotherapy, which may negate the functional and cosmetic benefits of the graft. In this series, 5 out of 15 cases with malignant pathology underwent postoperative radiotherapy and radiotherapy was shown to have no impact on postoperative incidence of Frey’s syndrome and facial contour.

Another reason for hesitation in reconstructing parotidectomy defects for malignant pathology is the fact that postoperative surveillance may be hindered by the graft as any nodularity and the bulk of the graft can be confused for recurrence on clinical exam. DFFG does not seem to hinder postoperative surveillance if imaging is used for surveillance. However, if imaging is not readily available, DFFG can hinder surveillance. At our institutions, grafted patients are followed with serial Ultrasound, CT, or MRI scan. These imaging modalities give excellent definition between the remaining parotid gland and the graft.^27^

## Limitations

One limitation of this study is the lack of a control group to compare cosmetic and functional outcomes in similar patients who did not undergo DFFG reconstruction. To claim that free DFFG is superior to other reconstruction techniques, future case-control studies could be designed. In this study, the patients were not assessed for presence or absence of comorbid conditions such as smoking, diabetes, and vascular diseases. These comorbidities may have an impact on the success of DFFG as they may inhibit neovascularization and tissue integration. In the study conducted by Honeybrook et al^28^, 2 out of 3 patients who demonstrated post-operative complications (infection, graft necrosis, and seroma), had such comorbid conditions.

Another limitation to this study is the short follow up for certain patients and the lack of consistency in the timing of questionnaire administration. The rate of reported facial asymmetry may be higher shortly after parotidectomy. On the other hand, Frey’s syndrome may also present as late as 6 months to 1-year post parotidectomy.^12^

Moreover, the 2 questionnaires for postoperative outcomes were not administered at the same time leading to a significant variation in the response rate. Initially, the study was not designed to evaluate outcomes at the abdominal graft donor site and the first cohort of the patients did not receive the questionnaire that addresses these outcomes. On further follow up, it was noticed that it is an important outcome that must be followed up and reported. Unfortunately, by the time the second questionnaire was designed, many patients had been lost to follow up, and only the second cohort of patients responded to both questionnaires.

## Conclusion

Immediate reconstruction of post-parotidectomy defects with autologous abdominal free DFFG effectively preserves facial cosmesis, prevents Frey’s syndrome and prevents the risks of secondary reconstruction. This surgical technique is simple, safe, tailored to the size of the defect, and is associated with minimal postoperative complication and minimal donor site morbidity. It can be used successfully for both malignant and benign pathology and in patients who require adjuvant radiation therapy to the parotid bed, although this needs to be confirmed in larger series.
